# Supplementary material for: Association of the rs8720 and rs12587 KRAS Gene Variants with Colorectal Cancer in a Mexican Population and Their Analysis In Silico
Source: Cells. 2023 Jul 26;12(15):1941. doi: 10.3390/cells12151941 (PMC10417115; doi:10.3390/cells12151941)
Supplement: Supplementary file 1 [file cells-12-01941-s001.zip › cells-2506157-supplementary.pdf]

Supplementary Materials:
Figures S1. Expression analysis of KRAS miRNA

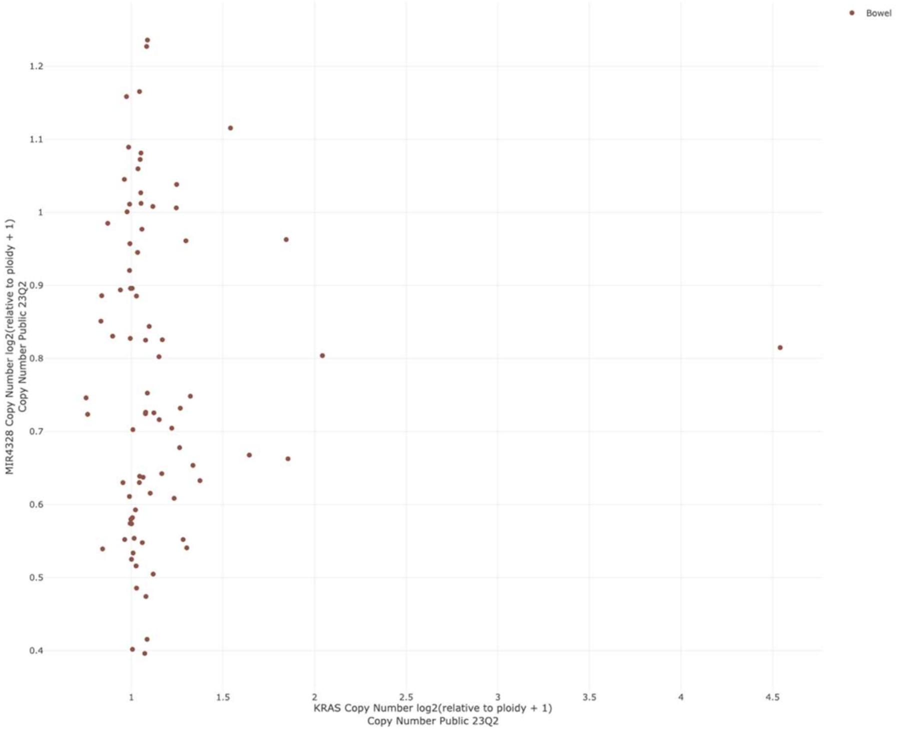

Figure S1-A. mKRAS/hsa-miR4328* (r=0.018, p=0.871).

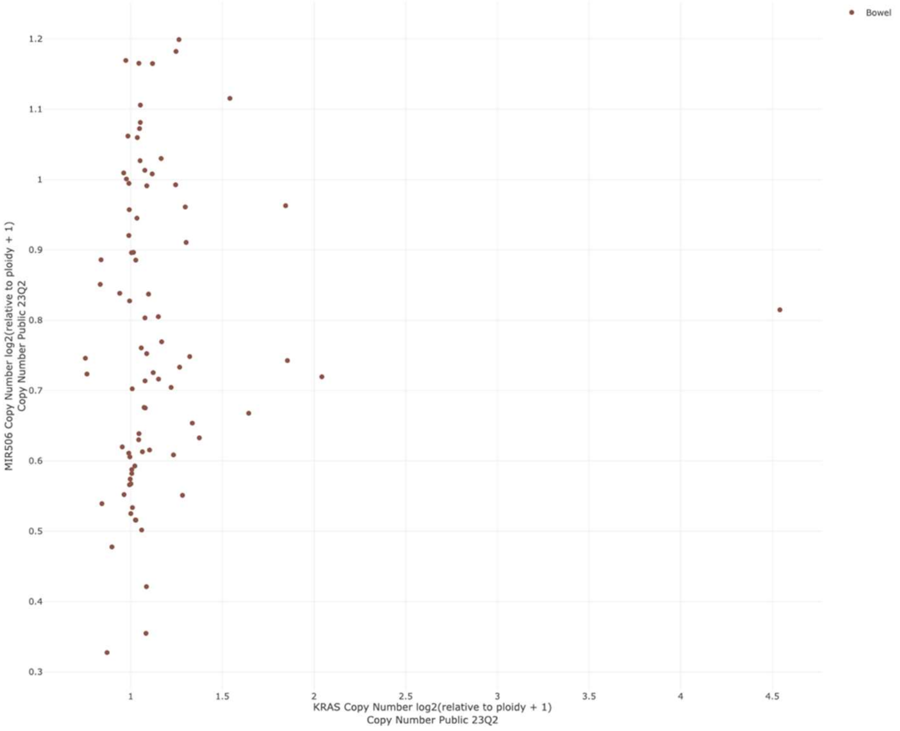

Figure S1-B. mKRAS/hsa-miR-506-50 (r=0.071, p=0.526).

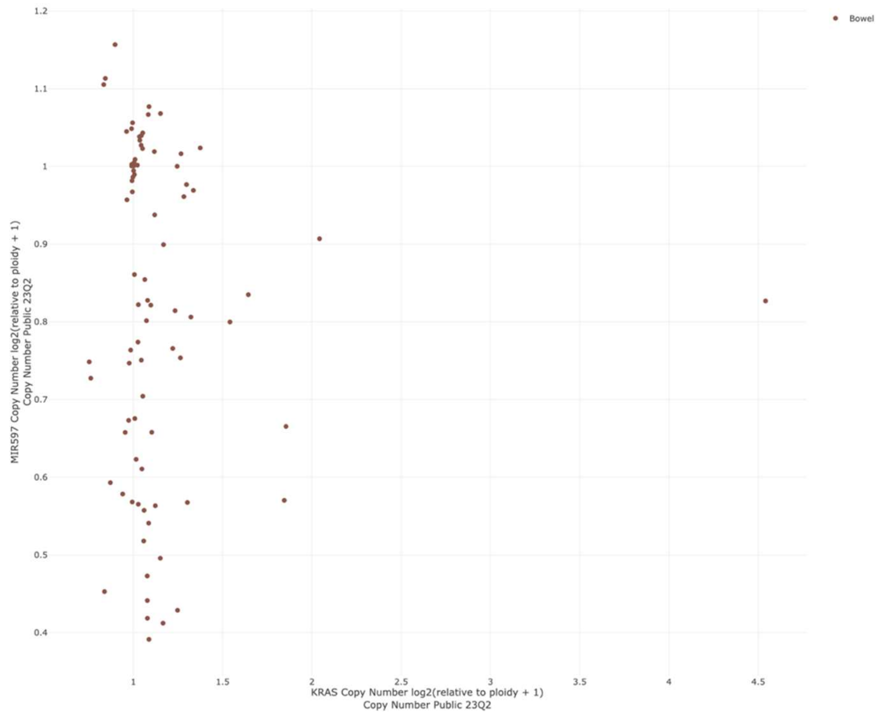

Figure S1-C. mKRAS/hsa-miR-597-3p (r=-0.037, p=0.743).

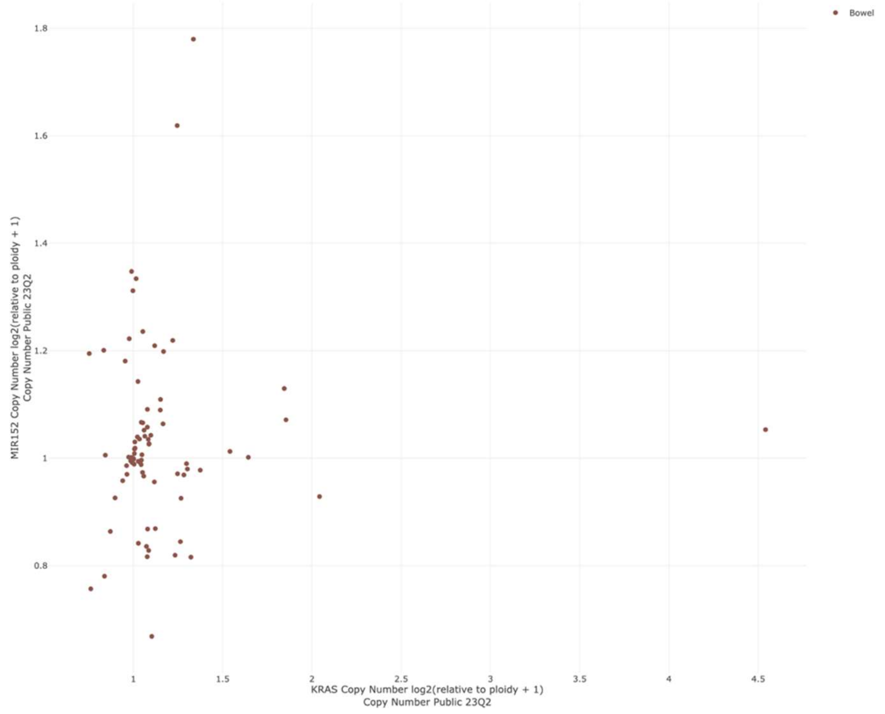

Figure S1-D. mKRAS/hsa-miR-152-5p (r=0.046, p=0.680).

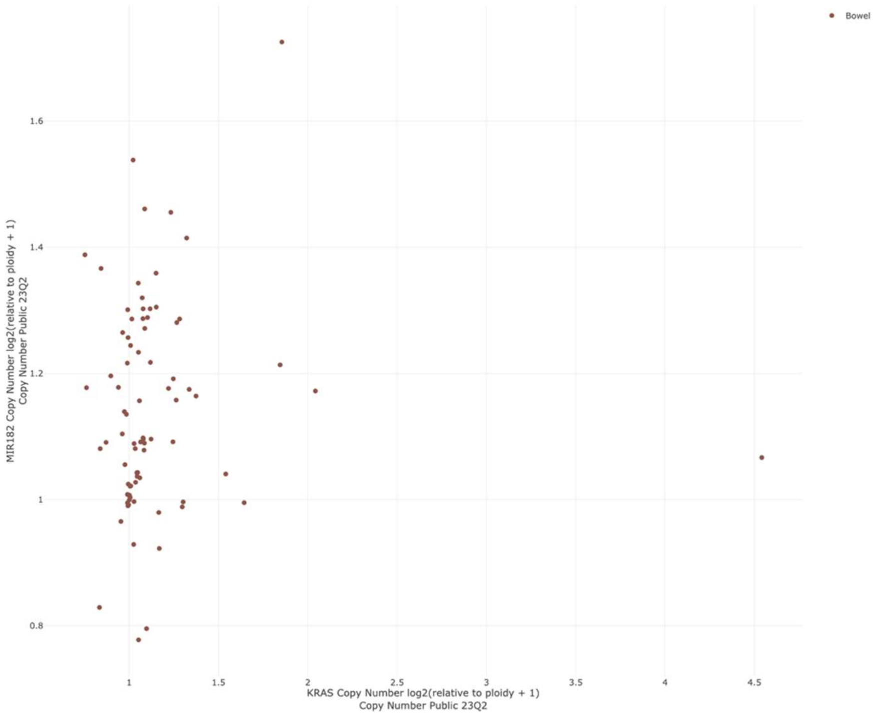

Figure S1-E. mKRAS/hsa-miR-182-3p (r=0.057, p=0.613).

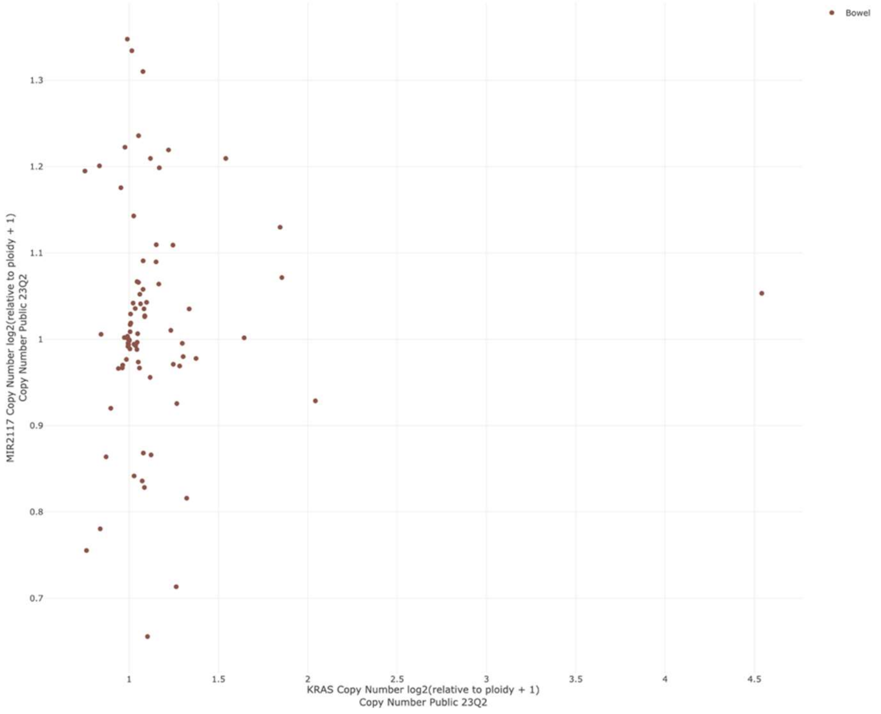

Figure S1-F. mKRAS/hsa-miR-2117 (r=0.040, p=0.724).

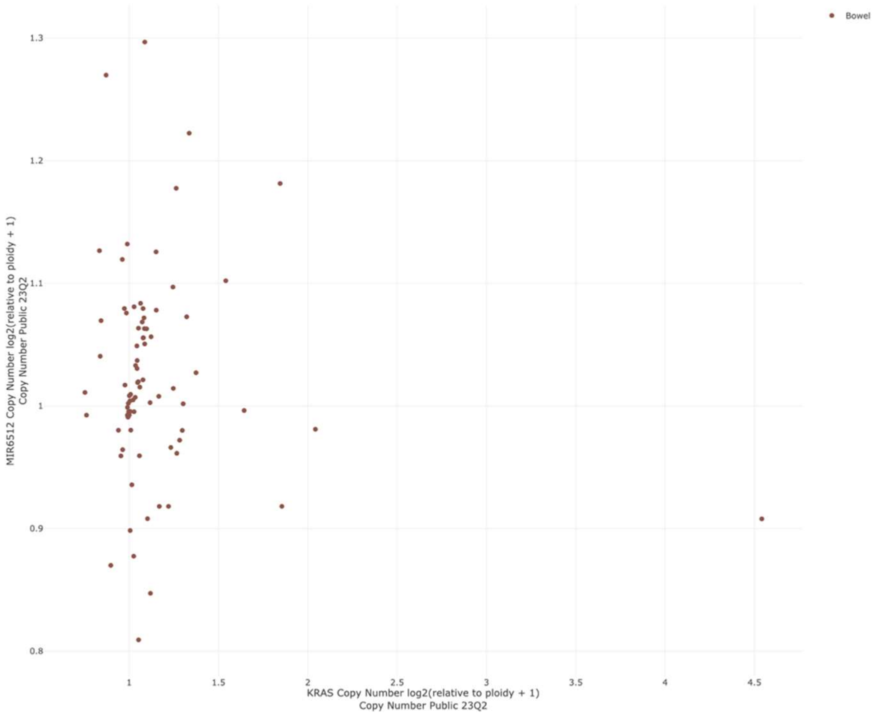

Figure S1-G. mKRAS/hsa-miR-6512-5p (r=-0.116, p=0.298).

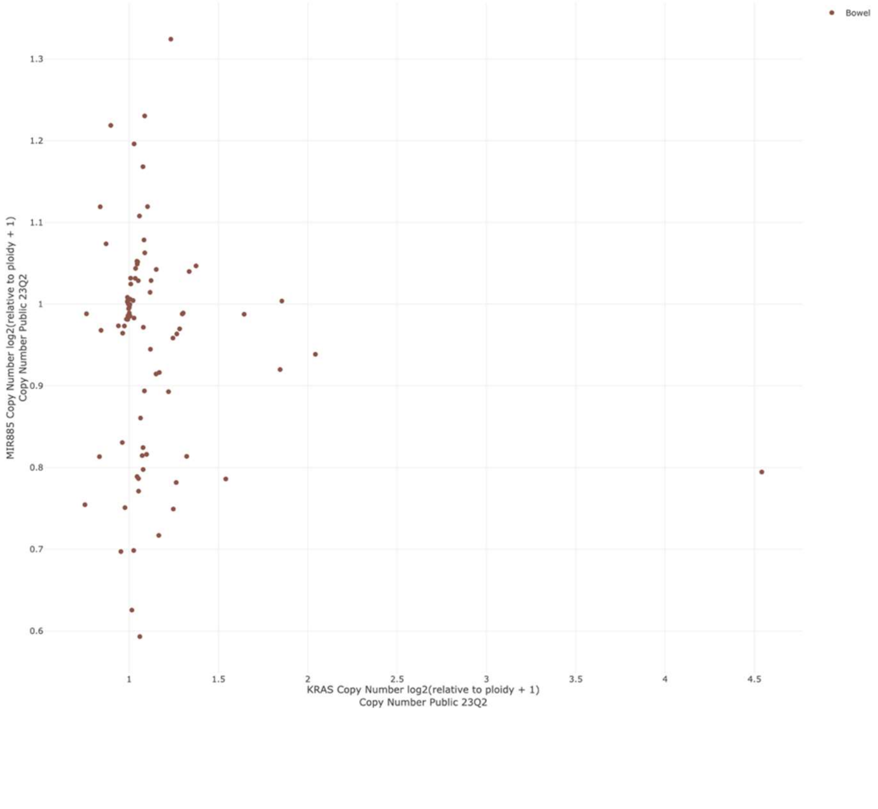

Figure S1-H. mKRAS/hsa-miR-885-5p (r=-0.124, p=0.269).

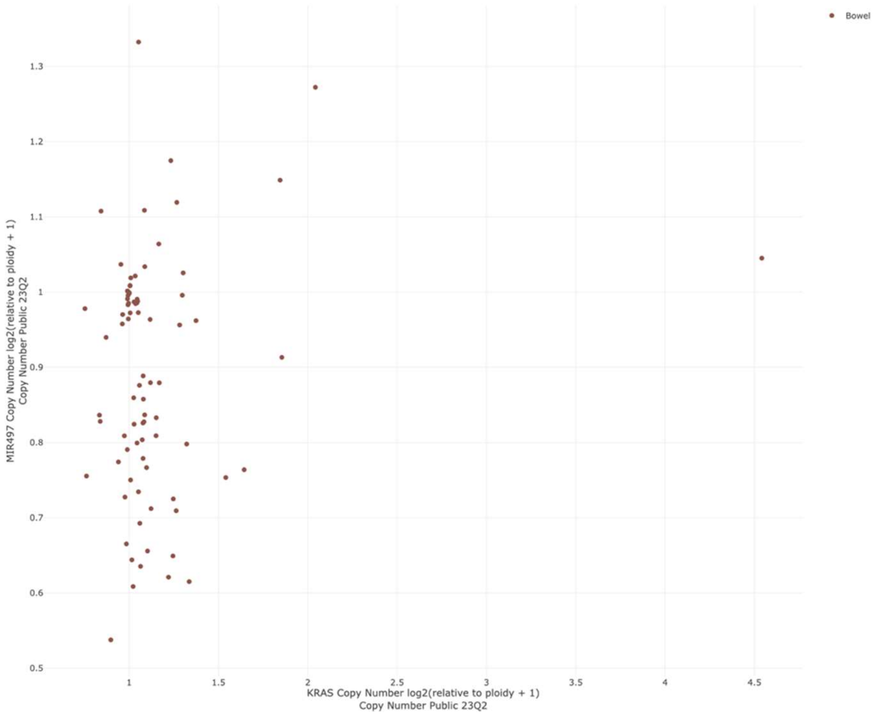

Figure S1-I. mKRAS/hsa-miR-497-3p (r=0.162, p=0.145).

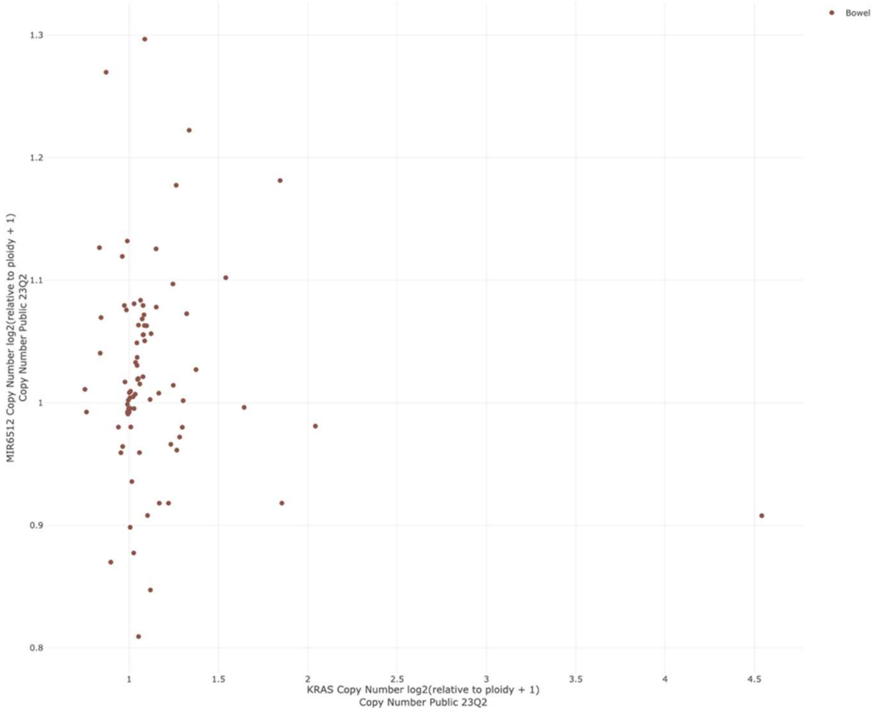

Figure S1-J. mKRAS/hsa-miR-6512-5p (r=-0.116, p=0.298).

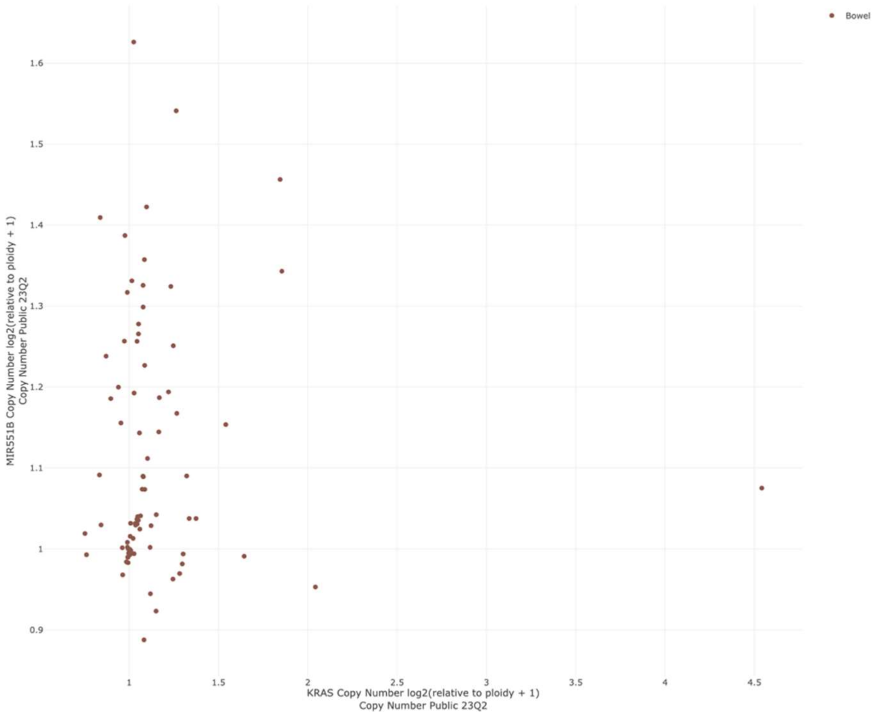

Figure S1-K. mKRAS/hsa-miR-551b-5p (r=0.023, p=0.835).

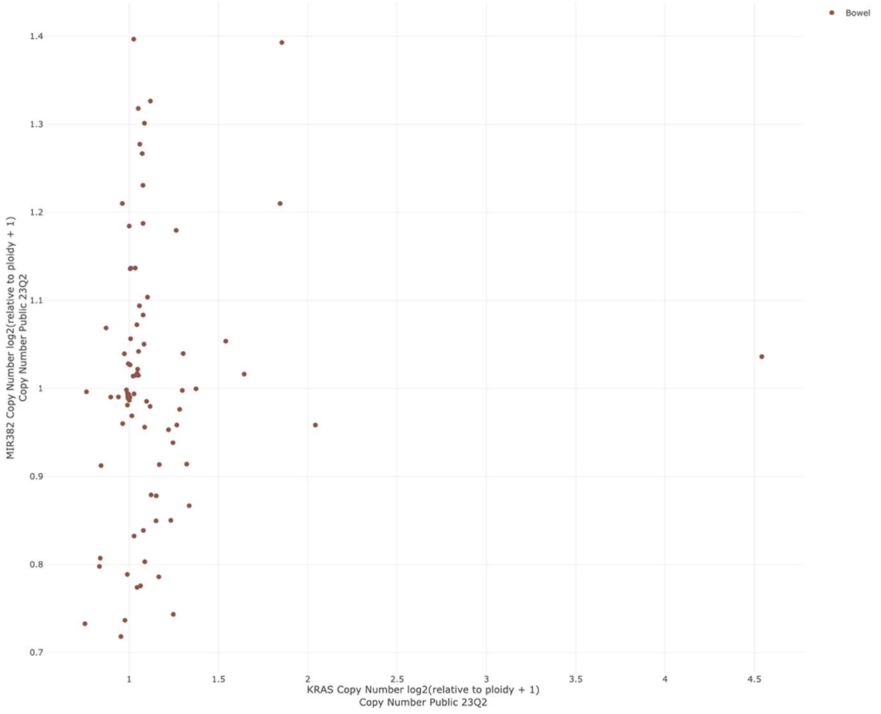

Figure S1-L. mKRAS/hsa-miR-382-3p (r=0.109, p=0.329).

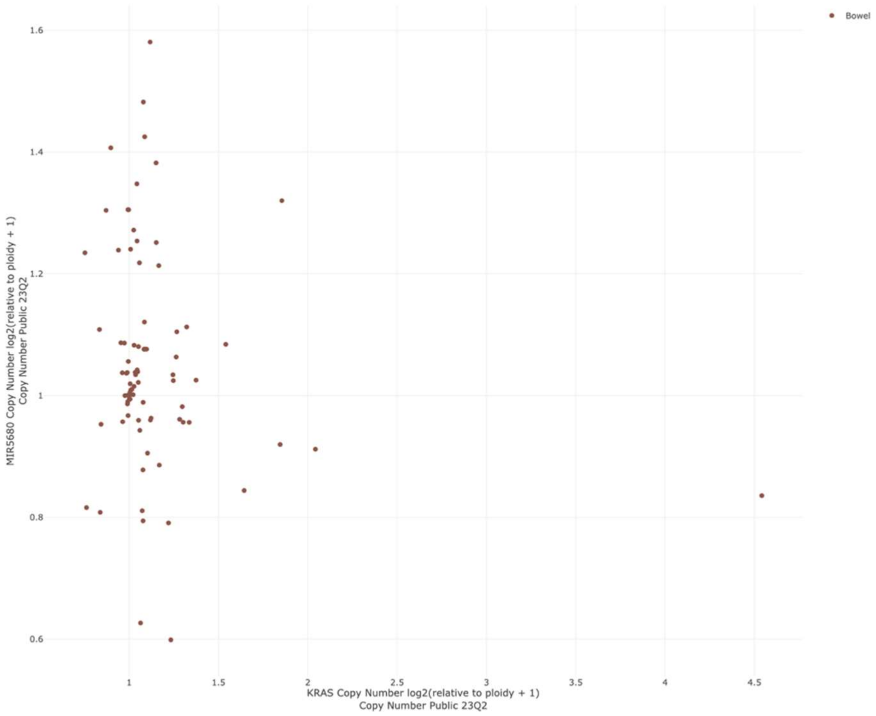

Figure S1-M. mKRAS/hsa-miR-5680 (r=-0.173, p=0.121).
